# Simultaneous Double-Vessel Coronary Thrombosis with Sudden Cardiac Arrest as the First Manifestation of COVID-19

**DOI:** 10.3390/medicina60010039

**Published:** 2023-12-25

**Authors:** Radojka Jokšić-Mazinjanin, Nikolina Marić, Aleksandar Đuričin, Marija Bjelobrk, Snežana Bjelić, Miloš Trajković, Mila Kovačević

**Affiliations:** 1 Faculty of Medicine, University of Novi Sad, 21000 Novi Sad, Serbia; aleksandar.djuricin@mf.uns.ac.rs (A.Đ.); marija.bjelobrk@mf.uns.ac.rs (M.B.); snezana.bjelic@mf.uns.ac.rs (S.B.); mila.kovacevic@mf.uns.ac.rs (M.K.); 2 Institute for Emergency Medical Services Novi Sad, 21000 Novi Sad, Serbia; maric1992@gmail.com; 3 Institute for Cardiovascular Diseases of Vojvodina Sremska Kamenica, 21208 Novi Sad, Serbia; milos.t.trajkovic@gmail.com

**Keywords:** COVID-19, out-of-hospital cardiac arrest, coronary thrombosis, percutaneous coronary intervention

## Abstract

The relationship between coronavirus disease 2019 (COVID-19) and myocardial injury was established at the onset of the COVID-19 pandemic. An increase in the incidence of out-of-hospital cardiac arrest was also observed. This case report aims to point to the prothrombotic and proinflammatory nature of coronavirus infection, leading to simultaneous coronary vessel thrombosis and subsequently to out-of-hospital cardiac arrest. During the COVID-19 pandemic, a 46-year-old male patient with no comorbidities suffered out-of-hospital cardiac arrest (OHCA) with ventricular fibrillation as the first recorded rhythm. The applied cardiopulmonary resuscitation (CPR) measures initiated by bystanders and continued by emergency medical service (EMS) resulted in the return of spontaneous circulation. The stabilized patient was transferred to the tertiary university center. Electrocardiogram (ECG) revealed “lambda-like” ST-segment elevation in DI and aVL leads, necessitating an immediate coronary angiography, which demonstrated simultaneous occlusion of the left anterior descending (LAD) and right coronary artery (RCA). Primary percutaneous coronary intervention (PCI) with the implantation of one drug-eluting stent (DES) in LAD and two DES in RCA was done. Due to the presence of cardiogenic shock (SCAI C), an intra-aortic balloon pump (IABP) was implanted during the procedure, and due to the comatose state and shockable cardiac arrest, targeted temperature management was initiated. The baseline chest X-ray revealed bilateral interstitial infiltrates, followed by increased proinflammatory markers and a positive polymerase chain reaction (PCR) test for severe acute respiratory syndrome coronavirus 2 (SARS-CoV-2) demasking underlying COVID-19-related pneumonia. Within the following 48 h, the patient was hemodynamically stable, which enabled weaning from IABP and vasopressor discontinuation. However, due to the worsening of COVID-19 pneumonia, prolonged mechanical ventilation, together with antibiotics and other supportive measures, was needed. The applied therapy resulted in clinical improvement, and the patient was extubated and finally discharged on Day 26, with no neurological sequelae and with mildly reduced left ventricle ejection fraction.

## 1. Introduction

Ever since the pandemic outbreak, it has been observed that coronavirus disease 2019 (COVID-19) has been involved in many sudden cardiac arrests (SCAs), with a significant increase in the incidence of SCA and cardiopulmonary resuscitations (CPRs) with unfavourable outcomes. According to the report from the Fire Department in Houston, Texas, the number of phone calls for SCAs, which resulted in an unfavourable outcome, increased by 45% [1]. Similarly, a two-times increase in out-of-hospital cardiac arrest incidences in Paris, France and Italy (50% and 58%, respectively), coupled with a reduction in survival rate, were also observed during the pandemic period [2,3].

At the onset of the epidemic in China, the relation between COVID-19 and myocardial injury with an increase in troponin levels was established. According to Shi S et al., among 416 hospitalized patients, 19.7% had myocardial injury and positive findings from their coronary angiography, which resulted in a higher mortality rate as compared to patients without myocardial injury [4].

Furthermore, it was described that up to 40% of hospitalized patients had elevated cardiac biomarkers, including troponin and brain natriuretic peptides, which have been linked to higher rates of in-hospital death [5,6].

Several reports point to the relationship between COVID-19 and acute coronary syndrome (ACS). 

In the Italian region of Lombardia, ST-segment elevation myocardial infarction (STEMI) was confirmed in 28 patients with COVID-19. In this group, 24 patients (85.7%) with STEMI as the first manifestation of COVID-19 were not positive on the COVID-19 tests at the moment of coronary angiography [7]. In the study from six New York hospitals, STEMI was observed in 18 patients with COVID-19. In two patients from the study group, SCA due to coronary artery thrombosis with STEMI was the first manifestation of COVID-19 [8]. In a multicenter international registry, COVID-19-positive ACS patients presented later and had increased in-hospital mortality compared to a pre-COVID-19 ACS population. The higher rate of cardiogenic shock contributed to worse outcomes in COVID-19-positive STEMI patients [9].

The relation between SCA due to coronary artery thrombosis and COVID-19 is still not thoroughly explained. However, the pathophysiological mechanism of coronary artery thrombosis is thought to include an enhanced systemic inflammatory response and a hypercoagulable state, leading to endothelial inflammation, endothelial injury and dysfunction and microcirculation damage [10].

This report aims to present a case of simultaneous double-vessel coronary thrombosis with out-of-hospital-cardiac arrest (OHCA) as the first manifestation of COVID-19 in a previously healthy patient without risk factors, comorbidities and with no family history of sudden cardiac death (SCD).

## 2. Case Report

**Figure 1:**
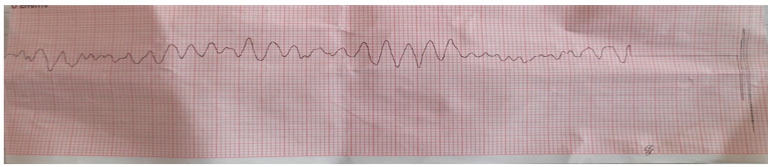
The initial rhythm on the cardiac defibrillator.

**Figure 2:**
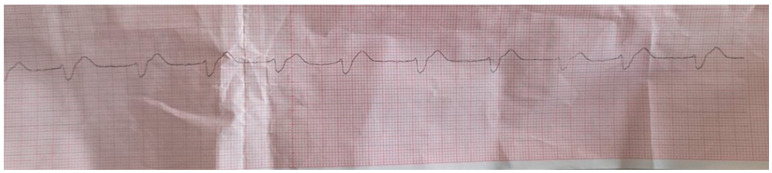
The rhythm after defibrilation and the two-minute CPR.

A phone call was received at the emergency medical services (EMS) from a bystander who reported a case of a 46-year-old male who lost consciousness and had no breathing. The EMS team arrived in 4 min. The patient was found lying on the bed, and two bystanders initiated CPR. The patient was unconscious, with no breathing and no pulse, with mydriatic unresponsive pupils. The initial rhythm monitored on the cardiac defibrillator was ventricular fibrillation (Figure 1), so a 150 J DC shock was delivered, and CPR was immediately resumed. The patient was endotracheally intubated and ventilated with a bag valve mask, and oxygen was administered at a flow rate up to 15 L per minute. After the two-minute CPR, pulseless electrical activity (PEA) was registered on the cardiac defibrillator (Figure 2). CPR was continued, and adrenaline (1 mg/mL) was administered every three minutes. After the third cycle of CPR and the third dose of adrenalin, VF was observed on the cardiac defibrillator. Two 200 J DC shocks were delivered (with a two-minute interval), followed by a 300 mg bolus of amiodarone. Finally, it resulted in the return of spontaneous circulation, and atrial fibrillation with HR of 120 bpm was observed. The patient was transferred to the Intensive Cardiac Care Unit (ICCU) of the tertiary University Cardiology Clinic.

**Figure 3:**
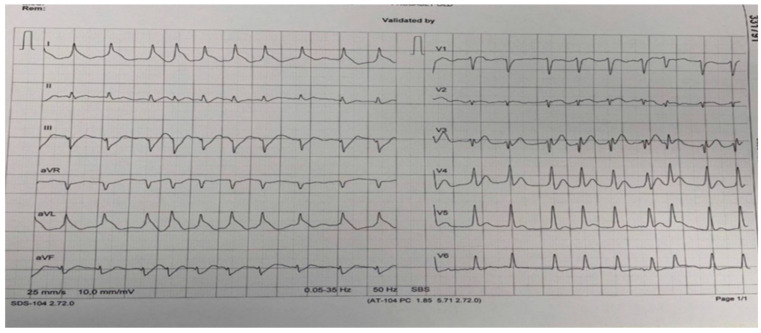
The admission ECG.

The medical history obtained from the patient’s family revealed no previous comorbidities, no risk factors and no familly history of SCD. However, on the day of the event, one hour prior SCA, he suffered some chest discomfort. At the admission to the ICCU, the patient was comatose (Glasgow Coma Scale, GCS = 7), hypotensive (BP 80/50 mmHg), tachycardic (HR 115 bpm), in atrial fibrillation, with elevated lactate in the blood gas analysis (lactate 3.4 mmol/L) and in cardiogenic shock (SCAI class C). The electrocardiogram (ECG) revealed a slight elevation in precordial leads and an uncommon ECG pattern called “lambda-like” ST-segment elevation in DI and aVL leads (Figure 3). The focus cardiac ultrasound (FoCUS) depicted a reduced ejection fraction (EF of 25–30%) with apical, anteroseptal and anterolateral akinesia and inferior hypokinesia, with no mechanical complication of MI. 

**Figure 4:**
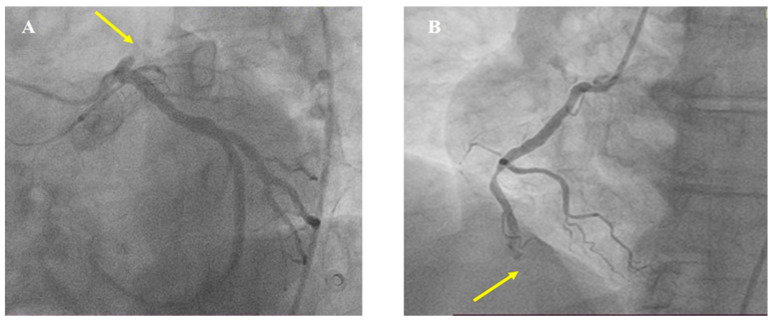
Baseline coronary angiography. (**A**) Left coronary artery, with proximal left anterior descending occlusion (arrow). (**B**) Right coronary artery, with distal occlusion (arrow).

An immediate coronary angiography was performed revealing the acute occlusion of two vessels, proximal left anterior descending (LAD) and distal right coronary artery (RCA), type B2, with Thrombolysis In Myocardial Infarction (TIMI) flow 0 (Figure 4).

**Figure 5:**
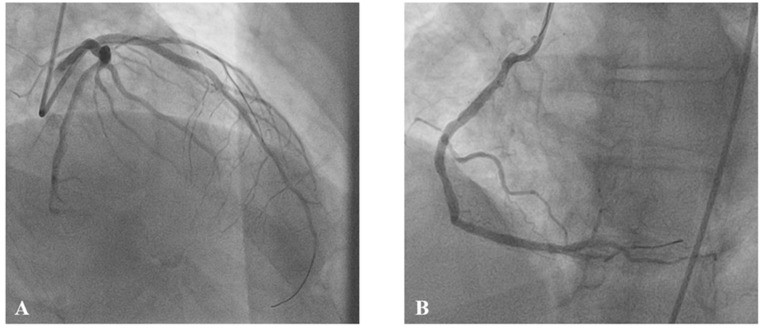
Coronary angiography after PCI. (**A**) Left coronary artery after PCI with one DES. (**B**) Right coronary artery after PCI with two DES.

The patient underwent primary percutaneous coronary intervention (PCI). Both coronary arteries were recanalized with a workhorse coronary guidewire (Sion Blue S). After the LAD wiring, TIMI flow I was established with apparent thrombus formation, TIMI thrombus grade (TG) 4. Due to the unsuccessful thrombus aspiration, predilatation with a semi-compliant (SC) balloon 3.0 × 15 mm was done, followed by a 3.5 × 38 mm drug-eluting stent (DES) implantation. After the recanalization of the LAD, RCA PCI was conducted. After the wiring of RCA, there was no coronary flow, with apparent TIMI TG 5. Therefore, thrombus aspiration was performed, which resulted in the aspiration of a small number of thrombi and, subsequently, in the restoration of the coronary flow. This was followed by lesion predilatation with a 3.5 × 15 mm SC balloon. Due to the large thrombus burden, Tirofiban was introduced intracoronary as well. Finally, two DESs 3.5 × 28 mm and 3.5 × 16 mm were implanted. The final angiographic result in both coronary arteries was optimal, with a final TIMI III flow (Figure 5). Due to the evident cardiogenic shock (SCAI C), an intraaortic balloon pump was implanted (IABP) during the procedure. 

**Figure 6:**
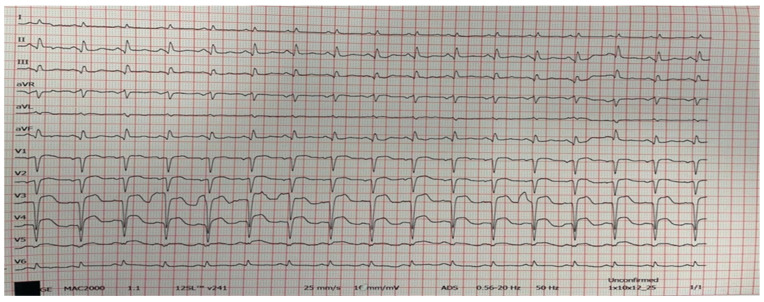
ECG after PCI and cardioversion.

**Figure 7:**
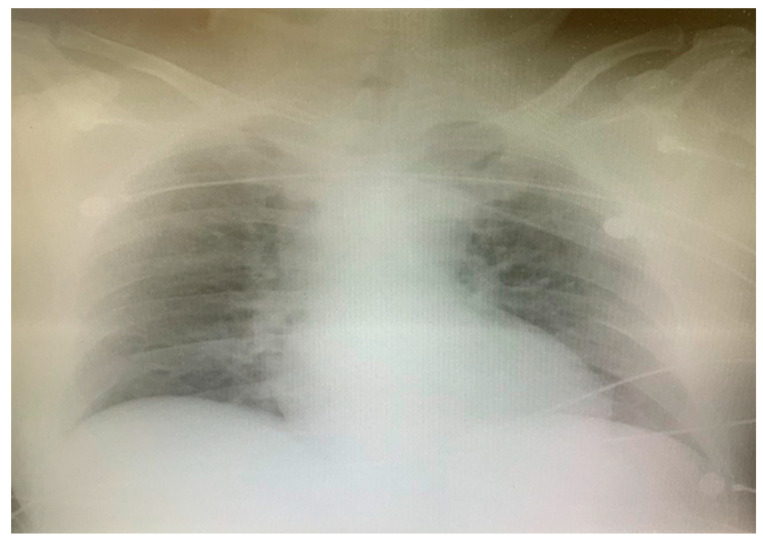
The admission chest X-ray.

After the primary PCI, the patient underwent cardioversion, and the sinus rhythm was restored (Figure 6). Due to OHCA with an initial shockable rhythm and comatose state (GCS 7), therapeutic hypothermia was initiated with the targeted temperature of 36 °C in the following 24 h. The admission chest X-ray revealed the interstitial opacities of inflammatory etiology (Figure 7). On Day 2, blood tests showed an increase in proinflammatory markers (leucocytes and CRP-C reactive protein) and D-dimer, while the PCR test for severe acute respiratory syndrome coronavirus 2 (SARS-CoV-2) turned out to be positive (Table 1). According to heteroanamnestic data received from the patient’s family, the patient did not receive any vaccine for COVID-19.

The complete blood count, inflammatory markers, myocardial necrosis biomarkers and hepatic and renal function markers were assessed through serial blood sampling (Table 1). 

In the following two days, the patient was hemodynamically stable, in sinus rhythm with a normal blood gas analysis and with normal urine output (>1 mL/kg/h). This enabled weaning from IABP 48 h after the PCI and noradrenalin discontinuation on Day 4. Echocardiography revealed improvement in the ejection fraction, which was estimated at 45% with apical akinesia and anteroseptal hypokinesia. 

**Figure 8:**
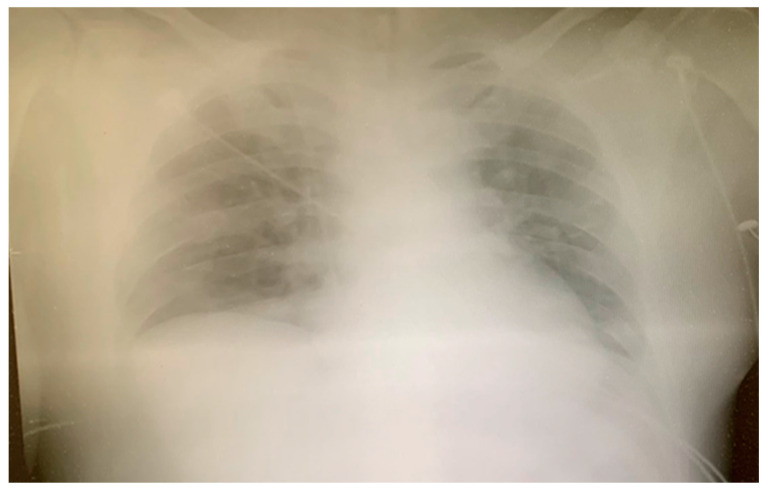
Chest X-ray on Day 7.

However, despite the introduction of parenteral antibiotics and other supportive measures, a progression of pneumonia was noticed on the serial chest X-rays (Figure 8), prohibiting weaning from mechanical ventilatory support.

Therefore, due to the worsening of SARS-CoV-2 pneumonia, the patient was transferred to the specialized COVID unit at the Institute for Pulmonary Diseases on Day 7. The patient was treated according to the current COVID-19 protocol. A concomitant Acinetobacter baumannii complex infection was found in the tissue specimen obtained by bronchoalveolar lavage. The intensive treatment of COVID-19 pneumonia was continued, resulting in improved clinical status. The patient was extubated on Day 15 with complete neurological recovery. He was alert and communicative, haemodynamically stable, in sinus rhythm, with a normal blood gas analysis, no signs of respiratory failure and no need for oxygen therapy, and finally, he was discharged on Day 26. 

On the three-month and one-year follow-ups, the patient was without chest pain and with no limitations on ordinary physical activity; the echocardiography showed an EF of 50%. 

## 3. Discussion

Cardiovascular complications are frequent in patients with COVID-19. 

The hypercoagulable state and systemic inflammation observed in COVID-19 are unique features causing myocardial injury, which can manifest either as type 1 or type 2 myocardial infarction (MI) or acute non-ischemic myocardial injury such as myocarditis, Takotsubo cardiomyopathy or acute heart failure [10,11].

In a report from the Italian region of Lombardia, 28 study group patients were treated for both STEMI and COVID-19 during February/March 2020. In 24 patients, the first diagnosis was STEMI, which also proved to be the first manifestation of COVID-19, indicating a significant correlation between coronary thrombosis and COVID-19 [7]. Multiple coronary thrombosis as the first manifestation of COVID-19 was also described in other studies [12,13].

A massive systemic inflammatory reaction associated with severe pneumonia, as in COVID-19, may lead to a local increase in inflammatory cells in coronary arteries, contributing to a higher atherosclerotic plaque vulnerability and, subsequently, to an increased tendency for plaque disruption [14] and thrombus formation, leading to type 1 MI [11].

Furthermore, higher rates of multiple coronary thrombosis, stent thrombosis and higher thrombus burden with no-reflow phenomenon have been reported in STEMI patients with COVID-19 as compared to those noninfected, altogether with a higher troponin level, D-dimer and CRP [15].

In our case report, OHCA was the first manifestation of type 1 MI and COVID-19 as well. After the ROSC was established, an uncommon ECG pattern, “lambda-like” ST-segment elevation, was observed. It is described as a warning sign for the development of VF in myocardial ischemia [16,17] and also reflects the presence of a large area of transmural myocardial ischemia and can predict cardiogenic shock associated with high in-hospital mortality [18]. In our case report, “lambda-like” ST-segment elevation implied a large area of jeopardized myocardium caused by simultaneous double-vessel occlusion. 

STEMI caused by the simultaneous thrombosis of two or more coronary arteries has been reported to occur in approximately 2.5% of patients undergoing primary PCI [19]. However, the exact incidence rate of “multiple culprit” arteries is difficult to establish, because most patients experience SCD, and according to autopsy findings, it can be seen in up to 50% of patients with SCD [20]. Our patient experienced OHCA as the first manifestation of MI, and due to the prompt reaction of bystanders and EMS, ROSC was established, and the diagnosis of STEMI caused by simultaneous coronary thrombosis was confirmed. 

The underlying mechanism of such a clinical presentation and simultaneous coronary thrombosis can be explained by the prothrombotic, procoagulant and mainly proinflammatory nature of SARS-CoV-2 infection, leading to endothelial inflammation, endothelial injury and dysfunction, microcirculation damage and a higher rate of atherosclerotic plaque rupture [10,14].

COVID-19 increases the risk of arterial and venous thrombosis, with raised levels of proinflammatory markers and D-dimer as well. However, plasma D-dimer levels reflect the activity of blood coagulation and fibrinolysis and also correlate with the duration of cardiac arrest, especially in OHCA patients due to cardiovascular causes [21]. Furthermore, D-dimer ≤ 10 μg/mL was found as an independent predictor for 30-day survival in patients with OHCA [21].

Apart from elevated high-sensitive cardiac troponin I, brain natriuretic peptide levels, lactate dehydrogenase and Il-6, which may have prognostic implications [6,22], lymphopenia may be considered as a cardinal laboratory finding with prognostic potential [23]. It has been reported that patients with severe disease and fatal outcomes present with a decreased lymphocyte/white blood cell ratio both on admission and during hospitalization [23,24]. Furthermore, contrary to non-survivors, the survivors demonstrated the nadir of the lymphocyte count on Day 7 from symptom onset and subsequent restoration [22].

The patient from our case report had significantly increased levels of D-dimer, mainly as a consequence of prolonged CPR, although the SARS-CoV-2 contribution cannot be excluded. Additionally, high levels of cardiac troponin I, observed in our patient, mainly contribute to acute MI. However, prolonged lymphopenia in the complete blood count may indicate a potential severe clinical course of COVID-19 in our patient.

Patients with MI and COVID-19 infection have a more severe clinical course, are more likely to present with cardiogenic shock and have worse 30-day mortality [15,25,26]. The higher rate of life-threatening arrhythmias and the need to perform CPR is noticed in patients with MI and COVID-19 infection compared to patients with MI without COVID-19 infection [27]. An increase in out-of-hospital cardiac arrests (OHCA) and sudden deaths were also observed during the pandemic period [17].

In Sweden, the prepandemic survival rate of patients with ROSC after OHCA was 33.4%. After the COVID-19 outbreak, it decreased to 30.3% or even 25.9% in the group of patients with COVID-19. At discharge, the survival rate of patients with OHCA and COVID-19 was 0% [28]. This decline in the survival rates of patients with OHCA during the COVID-19 pandemic was reported in other studies as well. In New York, the prepandemic survival rate after OHCA was 25.2%, which fell to 10.6% during the pandemic [29]. In Northern Italy, a decrease of 8.5% was reported for the survival rate compared to the prepandemic period [3]. A US study comparing the outcomes of OHCA in the same months of the year, in the prepandemic period and during the COVID-19 outbreak, revealed that the ROSC rates decreased significantly during the pandemic. The decrease was even more significant in the states with moderate and high mortality rates from COVID-19 (higher than 100 or 500 deaths from COVID-19 per one million inhabitants, respectively) [30]. These findings indicate that the rate of patients with ROSC after OHCA decreases if associated with COVID-19. The survival rates during the hospitalization were also lower in patients with associated coronary disease and COVID-19. 

The patient from our case report had OHCA as the first manifestation of both STEMI and COVID-19. Due to the prompt reaction of bystanders and EMS, ROSC was established, allowing timely PCI, thus increasing the chances for survival. 

It is well established that survivors of cardiac arrest bear a substantial risk of neurological injury either via protracted hypoxia or reperfusion damage. Therefore, targeted temperature management (33–36 °C) was introduced to improve the neurological outcomes and survival. The comparative benefit of lower (32–34 °C) versus higher (36 °C) temperatures is still unknown. Furthermore, according to the latest evidence, there is no advantage in targeted hypothermia at 33 °C compared to targeted normothermia with preventing fever (body temperature > 37.8 °C) [31].

In our case resport, we conducted targeted hypothermia at 36 °C with an intravascular cooling device, with the main purpose of preventing fever. Timely resuscitation and targeted temperature management after OHCA due to a shockable rhythm in our patient resulted in complete neurological recovery. 

## 4. Conclusions

The COVID-19 pandemic significantly increased the incidences of myocardial injury and OHCA. Coronary thrombosis and OHCA may be the first manifestations of COVID-19. If associated, MI and COVID-19 significantly decrease the survival rates during hospitalization. However, the adequate and timely intervention of prehospital EMS, combined with a multidisciplinary approach during the hospitalization, improves the chances of survival, helping the recovery of patients with no cardiological or neurological complications. 

## Figures and Tables

**Table 1:** Serial blood sampling of inflammatory, cardial, hepatic and renal function markers, from Day 1 to Day 7.

	Reference Range	Day 1	Day 2	Day 3	Day 5	Day 6	Day 7
RBC	4–10 × 10^12^	4.49	5.01	4.6	4.0	4.0	3.72
Hb	140–180 g/L	139	157	143	123	123	115
WBC	4.00–10.00 × 10^9^	11.9	19.17	13.9	8.7	8.76	7.40
NEUT	2.00–7.00 × 10^9^	3.60	16.11	12.1	7.6	7.02	5.47
LYMPH	0.80–4.00 × 10^9^	7.75	1.22	0.9	0.6	0.75	0.79
PLT	150–450 × 10^9^	165	270	199	160	215	237
CRP	<8.0 mg/L	3.5	8.2	163	241	249.5	238.6
Glucose	3.3–6.1 mmol/L	12.5		8.7	7.7	6.8	7.0
Urea	2.8–7.2 mmol/L	5.8	8.5	10.7	7.4	6.7	10.4
Creatine	53–120 μmol/L	133	140	143	102	65	78
AST	7–55 U/L	137	1436	630	341	137	97
ALT	<45 U/L	214	440	334	257	142	104
LDH	241 U/L	311	2458	2396	1582	1076	817
Hs-cTnI	<34.2 ng/L	832	>50,000			18,091.2	
CK	55–170 U/L	217	4000	6321	2604	1219	538
CK-MB	<4.87 ng/mL	60	500	711	123	42	27
D-dimer	<500 ng/ml		>10,000	4702			3266

RBC—red blood cells; Hb—hemoglobin; WBC—leukocytes; NEUT—neutrophils; LYMPH—lymphocytes; PLT—platelets; CRP—C-reactive protein; AST—aspartate aminotransferase; ALT—alanine aminotransferase; LDH—lactate dehydrogenase; Hs-cTnI—high-sensitivity cardiac troponin I; CK—creatine kinase; CK-MB—creatine kinase MB.

## Data Availability

Data are contained within the article.
